# Dealing With Diversity in Psychology: Science or Ideology?

**DOI:** 10.1177/17456916241236170

**Published:** 2024-04-23

**Authors:** Bernhard Hommel

**Affiliations:** 1 Department of Psychology, Shandong Normal University; 2 Department of Psychology, TU Dresden

**Keywords:** race

## Abstract

The increasing use of political activist arguments and reasoning in scientific communication about diversity is criticized. Based on an article of Roberts et al. (2020) on “racial inequality in psychological research,” three hallmarks of the intrusion of activist thinking into science are described: blindness to the multidimensional nature of diversity, the failure to distinguish psychological mechanisms from the impact of moderators, and a blindness to agency as an explanation for psychological observations. It is argued that uncritically accepting and introducing political activist arguments into science is likely to damage scientific freedom and independence.

**Publisher’s Note**

This article is part of a collection of articles related to the December 2022 APS Vote of No Confidence in the Editor-in-Chief of *Perspectives on Psychological Science*. Please see the editorial [https://doi.org/10.1177/17456916241246556] in this issue for further details on the collection.

One of the strongest arguments for diversity can be derived from Darwin’s theory of evolution: If we would all be the same, think the same, and do the same, our species would be extremely vulnerable. Every slight change in our environment might be a potential threat, as it might render our strategy to deal with life ineffective and useless overnight. It is thus important that we are different, think differently, and do things differently. Appreciating that is hard, because it is much easier to communicate and get along with people like us than with people who are different, and this also holds for science. Accordingly, it is important to keep emphasizing the importance of diversity, and so we all should welcome the recent increase in interest in diversity and its importance. Yet discussions about the importance of diversity in science are often penetrated by ideology and activist thinking which is more interested in the benefit of one particular minority group than in diversity as such. An example in case is the recent article of Roberts et al. (2020) on “racial inequality in psychological research.” As I will explain, this article shows three of the most frequent and most worrying signs of ideological thinking and social justice activism that have made their way into science. That this is no exception is witnessed by the fact that all three signs are favorably echoed in the comment on this article by Dupree and Kraus (2022).

## Blindness to the Multidimensional Nature of Diversity

Political activist groups commonly focus on the one personal feature their members share or identify with, such as gender/sex, race, or sexual orientation, and try to attract societal attention to it. That seems effective and understandable, the more so as the same individual might be involved in different activist groups to attract societal attention to other possible features whenever needed. But if the same happens in science, justification is due. It is true that scientific research is always necessarily selective, and so one can always wonder whether that selectivity matters and perhaps limits possible theoretical insights and conclusions (Haeffel et al., 2009). But people, including potential participants in psychological studies, differ with respect to hundreds, if not thousands, of features. Many of them have been demonstrated to affect human cognition and behavior: sex/gender, race, culture (Arnett, 2009), religion (Hommel & Colzato, 2017), sexual orientation (Colzato, van Hooidonk, et al., 2010), socioeconomic background (Duncan & Magnuson, 2012), political orientation (Duarte et al., 2015), intelligence, motivational structure and needs (McClelland, 1988), learning history, upbringing, educational style, experience, personality and traits, body size, body weight, handedness, various kinds of disabilities, and cognitive styles, to name just a few. Some of them are likely to be interrelated in complex ways, such as race, economic background, and culture/country (e.g., racial differences correlate with economic differences in some countries more than in others), while other features may operate independently. Accordingly, it is important to consider the possibility that some of these features, or their interrelations, affect behavior in a way that is of theoretical relevance (Stroebe & Nijstad, 2009), perhaps even to a degree that would call for an adjustment or extension of theoretical models. That has been done in many studies, even though the type of features seems to underlie some seasonal changes, if not fashions, such as the great interest in societal background in the 1960s, the increasing interest in gender/sex in the 1970s, the increasing interest in ecological conditions in the 1980s, with a strong emphasis on race since the 2010s. One could always do more so that it makes sense to keep emphasizing further variables that might be interesting to study.

But this is not what Roberts et al. (2020) advocate. They instead focus on one single feature: race. They report data showing that this feature has been rarely addressed in developmental and social psychology and almost neglected in cognitive psychology, that most publications were edited by White editors, that most publications highlighting race were authored by White scientists, and that these employed fewer participants of color. It must be said that some of these findings are trivial and some more merely reflect the arbitrary design choices of the authors. For instance, for unclear (and unfortunate; see Arnett, 2009) reasons, the authors only consider journals from North American or European publishers, with authors mainly stemming from North America and Europe. The specific journal selection provides a source of confounds already: The two cognitive journals are issued by the Dutch private publisher Elsevier, whereas the two developmental and the two social journals are issued by U.S./American societies. Societies change their editors much more frequently than private publishers do (e.g., Jacques Mehler founded the journal *Cognition* and was chief editor from 1975 to 2007; Gordon Logan was chief editor of *Cognitive Psychology* from 1999 to 2021), and American and European publishers differ in their choices of editors (e.g., *Acta Psychologica*, another Elsevier journal, prefers chief editors with affiliation in Dutch-speaking countries), which is likely to account for some of the effects that Roberts et al. attribute to differences between research areas. Furthermore, Roberts et al. report to have only considered articles in which the term “race” or “racial” featured in the title and/or abstract. Given that both terms are considered to be scientifically obsolete (e.g., Cavanagh, 2019; Chen, 2016; Rice, 2009), Roberts et al. have missed articles of authors that for these or other reasons have presented their research on “racial” effects as studies of “ethnicity” or “cultural background” (e.g., Kearins, 1981; Ojalehto et al., 2017; Sparks et al., 2016; Wang, 2008).

Moreover, the asymmetries reported by Roberts et al. seem much less worrisome if appropriate base rates are considered. The percentage of people of color (POC) among the population amounts to about 38.4% in the United States (in 2020: Jones et al., 2021), 22% in Canada (in 2016: Statistics Canada, 2019), and around 10% in Europe, where in many countries no official numbers are collected (Banks, 2019). Considering the corresponding population sizes (329, 38, and 746 million, respectively), this amounts to an expected ratio of about 81% of White editors. This seems to compare rather well with the 87% White chief editors that Roberts et al. report, especially if one considers the much higher percentage of White citizens in the beginning of the time period the authors consider (1974–2018), who accounted for 87.7% (1970), 83.1% (1980), and 80.3% (1990) of the U.S. population (“Historical Racial and Ethnic Demographics of the United States,” 2022). With respect to the editorial board members, of which Roberts et al. found 76% to be non-POC, White researchers would even be slightly underrepresented. Moreover, given that White authors are more likely to come from countries with rather small percentages of POC citizens (< 10% in Germany, including only 1.4% from Africa: “Demographics of Germany,” 2022; approximately 10% in Europe; Banks, 2019), it is not overly surprising that POC participants are rare in their studies. And yet the authors conclude that strong efforts need to be made in order to increase racial diversity “in editing, writing, and participation” to achieve more representative distributions of race with respect to editorial positions, reviewers, authors, and participants.

It is not so much the unfounded nature of this request (given that the distribution is representative already) that concerns me here but the fact that the call for diversity was restricted to just one of more than 100 possible personal features of already demonstrated psychological relevance. If we are to report the race of editors, reviewers, authors, and participants, as Roberts et al. demand, it seems logical to also demand reporting their cultural, religious, and economic background; their political orientation, intelligence, and motivational structure; their needs, learning history, experience, and personality and traits; their body size, handedness, disabilities, and cognitive style; in addition to tens if not hundreds of other personal features that might have affected the measures of interest in past studies. And reporting would not be enough; the next scientifically meaningful step would be to try balancing all these features so that possible effects could be statistically estimated. The administrative and financial burden such demands imply would be enormous. But even the report of race (or ethnicity or cultural background), the single feature that Roberts et al. focus on, is more complicated than the authors seem to assume. If race is a social construct, as Roberts et al. suggest, there is no reason to believe that this construct is comparable across cultures or subcultures. For instance, it seems unlikely that, say, a Black individual raised in the United States is more similar to a Black individual raised in South Africa, Brazil, and China, say, than it is to a White individual raised in the United States. In any case, a closer look does not leave any scientific justification for the exclusive focus on race and the corresponding neglect of the many other personal features that we know can affect human behavior.

## Failure to Distinguish Mechanisms From Effect Moderators

Another flaw of the reasoning offered by Roberts et al. is the failure to distinguish between the mechanisms underlying a particular effect and the moderators of the size of this effect. While it is true that many features relating to individual differences were demonstrated to have an impact on human cognition and performance, including race, differences in effect sizes do not necessarily indicate different kinds of processes. For instance, there is evidence that religious faith has a systematic impact on the size of experimental effects in cognitive tasks that are considered to be indicative of basic processes: Members of individualistic religions tend to show less distraction in tasks that rely on attentional focusing than members of collectivistic religions (Hommel et al., 2011), whereas tasks that require integration of information show the opposite pattern (Colzato, Hommel & Shapiro, 2010). On the one hand, this clearly shows that religion does impact cognitive performance, and it may well be that race can also be shown to have an impact of that kind. On the other hand, however, that does not necessarily imply that the mechanisms at work are any different. Indeed, current theorizing assumes that societal factors can systematically increase or decrease effect sizes in basic tasks without any impact on the actual cognitive mechanisms (Hommel & Colzato, 2017). Given that cognitive sciences are interested in the mechanisms but not in absolute effect sizes or modulations thereof, it comes with little surprise that, as Roberts et al. observe, the cognitive sciences care less about social/ demographic diversity of the investigated participants. Even if they did, it is hard to see why and how that should limit the mechanistically relevant conclusions drawn from investigating participants without fully representative biographies. We do need to worry if there would be evidence that, say, POC and White participants differ systematically with respect to *how* they process distracting information, retrieve memories, and plan their actions. But not any such evidence is mentioned by Roberts et al.

## Blindness to Agency

A particularly salient feature of political activism in the recent years is the equation of nonrepresentative statistical distributions of features over positions or resources on the one hand and social injustice on the other (Sowell, 2019). If, thus, the distribution of race over editors, say, would really not match the distribution of race over the relevant societal reference group (which Roberts et al. fail to define), it is concluded that this reflects an inequality calling for societal worry (e.g., Cain Miller et al., 2018). Interestingly, this does not apply to all possible positions or resources: Few complain about the preponderance of females among hairdressers and of males among garbage disposers, but the more attractive, lucrative, and influential particular positions are, the more representativeness and participation becomes an issue. If then a particular position is particularly attractive, lucrative, and influential, and if the feature under discussion is not distributed representatively, the conclusion is that this must be due to structural societal obstacles, which need to be actively removed—a kind of thinking that Hughes (2018) has coined the “disparity fallacy.” This is indeed the idea that underlies the recommendations of Roberts et al., who (despite their data indicating racial representativeness) call for various kinds of actions to remove the assumed obstacles to POC in research.

Interestingly, this kind of reasoning reflects a strong bias toward one of (at least) two possible factors that decide whether an individual will occupy a particular position or indeed carry out any kind of action: intent (s/he wants it) and circumstances (which can enable or block the agent)—factors that roughly correspond to Heider’s (1958) motivation and capacity and Kelley’s (1973) personal and circumstance attributions. Unfortunately, the racial distribution analysis of Roberts et al. considers only circumstances as possible factors for uneven or nonrepresentative distributions, while intent is entirely dismissed. This seems to deny any possible impact of intent, any possible role of free choice. For no good reasons, I would argue, as there are neither logical nor empirical grounds to exclude that, say, individuals growing up under special conditions, such as members of a sizable minority in the United States (the country/culture Roberts et al. focus on almost exclusively) that were discriminated by law until the mid-1960s, develop different needs and interests than studying task switching or the automaticity of flanker processing in artificial laboratory experiments. Even if that were the case, this would neither constitute a societal problem nor would it need to be resolved. It would thus be crucial to consider data that might suggest that the selective engagement of POC in science, in psychology, and/or in particular psychological subdisciplines could reflect circumstances rather than intent and individual preferences. And yet, without presenting and discussing any such data, Roberts et al. jump from seemingly nonrepresentative distributions to the bold claim that there is something wrong with how psychological science is organized. I do not consider this a sufficiently balanced and a sufficiently scientific approach.

## Conclusion

It is important for science to be responsible and responsive to societal needs and developments. Therefore, studying political activism can be as useful as being stimulated by it (Conde, 2014), and by addressing questions that political activists are raising. And yet, uncritically accepting activist claims, demands, and reasoning and translating them into scientific practice creates a potentially toxic mix of science and ideology that is likely to damage scientific freedom and independence. True and scientifically justified interest in human diversity needs to consider all behaviorally relevant features in which we differ, to carefully distinguish between psychological mechanisms and moderators, and to consider both agency and circumstance in the explanation of human cognition and behavior.

## Transparency

*Action Editor:* Klaus Fiedler

*Editor:* Klaus Fiedler

## References

[bibr1-17456916241236170] Arnett J. J. (2009). The neglected 95%, a challenge to psychology’s philosophy of science. American Psychologist, 64(6), 571–574. 10.1037/a0016723

[bibr2-17456916241236170] Banks M. (2019, May 26). Representation of people of colour in EU elections is ‘abysmal’, says anti-racism group. The Parliament Magazine. https://www.theparliamentmagazine.eu/news/article/representation-of-people-of-colour-in-eu-elections-is-abysmal-says-antiracism-group

[bibr3-17456916241236170] Cain Miller C. Badger E. Hurd N. Kendi I. X. Hendren N. Chetty R . (2018, March 27). ‘When I see racial disparities, I see racism.’ Discussing race, gender and mobility. The New York Times. https://www.nytimes.com/interactive/2018/03/27/upshot/reader-questions-about-race-gender-and-mobility.html

[bibr4-17456916241236170] Cavanagh M. (2019, September 11). Don’t use the term ‘race’, German scientists urge. Deutsche Welle News. https://www.dw.com/en/dont-use-the-term-race-german-scientists-urge/a-50390582

[bibr5-17456916241236170] Chen A. (2016, February 5). Is it time to stop using race in medical research? NPR. https://www.npr.org/sections/health-shots/2016/02/05/465616472/is-it-time-to-stop-using-race-in-medical-research

[bibr6-17456916241236170] Colzato L. S. Hommel B. Shapiro K. (2010). Religion and the attentional blink: Depth of faith predicts depth of the blink. Frontiers in Psychology, 1, Article 147. https://doi.org/10.3389%2Ffpsyg.2010.00147 10.3389/fpsyg.2010.00147 PMC3153765 21833216

[bibr7-17456916241236170] Colzato L. S. van Hooidonk L. van den Wildenberg W. P. M. Harinck F. Hommel B. (2010). Sexual orientation biases attentional control: A possible gaydar mechanism. Frontiers in Psychology, 1, Article 13. 10.3389/fpsyg.2010.00013 PMC3095381 21607070

[bibr8-17456916241236170] Conde N. (2014). Activism mobilizing science. Ecological Economics, 105, 67–77.

[bibr9-17456916241236170] Demographics of Germany. (2022). In Wikipedia. https://en.wikipedia.org/wiki/Demographics_of_Germany#Ethnic_minorities_and_migrant_background_(Migrationshintergrund), retrieved 2/4/2024

[bibr10-17456916241236170] Duarte J. L. Crawford J. T. Stern C. Haidt J. Jussim L. Tetlock P. E. (2015). Political diversity will improve social psychological science. The Behavioral and Brain Sciences, 38, Article e130. 10.1017/S0140525X14000430 25036715

[bibr11-17456916241236170] Duncan G. J. Magnuson K. (2012). Socioeconomic status and cognitive functioning: Moving from correlation to causation. Wiley Interdisciplinary Reviews: Cognitive Science, 3, 377–386. 26301469 10.1002/wcs.1176

[bibr12-17456916241236170] Dupree C. H. Kraus M. W. (2022). Psychological science is not race neutral. Perspectives on Psychological Science, 17(1), 270–275. 10.1177/1745691620979820 33651963

[bibr13-17456916241236170] Haeffel G. J. Thiessen E. D. Campbell M. W. Kaschak M. P. McNeil N. M. (2009). Theory, not cultural context, will advance American psychology. American Psychologist, 64(6), 570–571. 10.1037/a0016191 19739896

[bibr14-17456916241236170] Heider F. (1958). The psychology of interpersonal relations. John Wiley & Sons. 10.1037/10628-000

[bibr15-17456916241236170] Historical racial and ethnic demographics of the United States. (2022). In Wikipedia. https://en.m.wikipedia.org/wiki/Historical_racial_and_ethnic_demographics_of_the_United_States, retrieved 2/4/2024

[bibr16-17456916241236170] Hommel B. Colzato L. S. (2017). The social transmission of metacontrol policies: Mechanisms underlying the interpersonal transfer of persistence and flexibility. Neuroscience and Biobehavioral Reviews, 81, 43–58. 28088534 10.1016/j.neubiorev.2017.01.009

[bibr17-17456916241236170] Hommel B. Colzato L. S. Scorolli C. Borghi A. M. van den Wildenberg W. P. M. (2011). Religion and action control: Faith-specific modulation of the Simon effect but not stop-signal performance. Cognition, 120, 177–185. 21546013 10.1016/j.cognition.2011.04.003

[bibr18-17456916241236170] Hughes C. (2018, May 14). The racism treadmill. Quillette. https://quillette.com/2018/05/14/the-racism-treadmill

[bibr19-17456916241236170] Jones N. Marks R. Ramirez R. Rios-Vargas M. (2021, August 12). U.S. Census Bureau. https://www.census.gov/library/stories/2021/08/improved-race-ethnicity-measures-reveal-united-states-population-much-more-multiracial.html

[bibr20-17456916241236170] Kearins J. M. (1981). Visual spatial memory in Australian Aboriginal children of desert regions. Cognitive Psychology, 13, 434–460. 7237993 10.1016/0010-0285(81)90017-7

[bibr21-17456916241236170] Kelley H. H. (1973). The process of causal attribution. American Psychologist, 28, 107–128.

[bibr22-17456916241236170] McClelland D. C. (1988). Human motivation. Cambridge University Press.

[bibr23-17456916241236170] Ojalehto B. L. Medin D. L. García S. G. (2017). Grounding principles for inferring agency: Two cultural perspectives. Cognitive Psychology, 95, 50–78. 28441519 10.1016/j.cogpsych.2017.04.001

[bibr24-17456916241236170] Rice M. (2009, February 11). Evolution and race: Biologically, race is no longer an issue, scientific panel agrees. Cornell Chronicle. https://news.cornell.edu/stories/2009/02/experts-biologically-race-no-longer-issue

[bibr25-17456916241236170] Roberts S. O. Bareket-Shavit C. Dollins F. A. Goldie P. D. Mortenson E. (2020). Racial inequality in psychological research: Trends of the past and recommendations for the future. Perspectives on Psychological Science, 15, 1295–1309. 10.1177/1745691620927709 32578504

[bibr26-17456916241236170] Sowell T. (2019). Discrimination and disparities. Basic Books.

[bibr27-17456916241236170] Sparks S. Cunningham S. J. Kritikos A. (2016). Culture modulates implicit ownership-induced self-bias in memory. Cognition, 153, 89–98. 27164187 10.1016/j.cognition.2016.05.003

[bibr28-17456916241236170] Statistics Canada. (2019). Data tables, 2016 Census. https://www12.statcan.gc.ca/census-recensement/2016/dp-pd/dt-td/Rp-eng.cfm?TABID=2&LANG=E&A=R&APATH=3&DETAIL=0&DIM=0&FL=A&FREE=0&GC=01&GL=-1&GID=1341679&GK=1&GRP=1&O=D&PID=110531&PRID=10&PTYPE=109445&S=0&SHOWALL=0&SUB=0&Temporal=2017&THEME=120&VID=0&VNAMEE=&VNAMEF=&D1=0&D2=0&D3=0&D4=0&D5=0&D6=0

[bibr29-17456916241236170] Stroebe W. Nijstad B. (2009). Do our psychological laws apply only to Americans? American Psychologist, 64(6), 569. 10.1037/a0016090 19739895

[bibr30-17456916241236170] Wang Q. (2008). Being American, being Asian: The bicultural self and autobiographical memory in Asian Americans. Cognition, 107, 743–751. 17920579 10.1016/j.cognition.2007.08.005

